# Inferring PDZ Domain Multi-Mutant Binding Preferences from Single-Mutant Data

**DOI:** 10.1371/journal.pone.0012787

**Published:** 2010-09-30

**Authors:** Elena Zaslavsky, Philip Bradley, Chen Yanover

**Affiliations:** 1 Center for Translational Systems Biology and Department of Neurology, Mount Sinai School of Medicine, New York, New York, United States of America; 2 Program in Computational Biology, Fred Hutchinson Cancer Research Center, Seattle, Washington, United States of America; Center for Genomic Regulation, Spain

## Abstract

Many important cellular protein interactions are mediated by peptide recognition domains. The ability to predict a domain's binding specificity directly from its primary sequence is essential to understanding the complexity of protein-protein interaction networks. One such recognition domain is the PDZ domain, functioning in scaffold proteins that facilitate formation of signaling networks. Predicting the PDZ domain's binding specificity was a part of the DREAM4 Peptide Recognition Domain challenge, the goal of which was to describe, as position weight matrices, the specificity profiles of five multi-mutant ERBB2IP-1 domains. We developed a method that derives multi-mutant binding preferences by generalizing the effects of single point mutations on the wild type domain's binding specificities. Our approach, trained on publicly available ERBB2IP-1 single-mutant phage display data, combined linear regression-based prediction for ligand positions whose specificity is determined by few PDZ positions, and single-mutant position weight matrix averaging for all other ligand columns. The success of our method as the winning entry of the DREAM4 competition, as well as its superior performance over a general PDZ-ligand binding model, demonstrates the advantages of training a model on a well-selected domain-specific data set.

## Introduction

Many vital cellular functions are mediated by protein complex formation [1]. Numerous such protein-protein interactions are enabled by peptide recognition domains, distinct structural units that bind specific amino-acid sequences in their interaction partners [1], [2]. Metazoan genomes encode dozens of peptide recognition domain families, each containing up to several hundred member proteins. Every family is typically characterized by a common fold and exhibits specificity to a particular ligand binding motif.

One important recognition domain is the PDZ domain, commonly found in organisms from bacteria to humans, and functioning in scaffold proteins to assemble large molecular complexes that facilitate formation of signaling networks [3], [4], [5]. The PDZ domain family is typically characterized by recognition of hydrophobic C-terminal tails, and individual members possess features that allow for distinct specificities within the broad structure and function of the family. Recent large-scale analyses of mouse [6] and human [7] data sets showed that PDZ-ligand interactions are highly specific, with distinct specificity classes evident among the binding motifs [7]. Moreover, this classification was found to be conserved throughout evolution.

Naturally, a question of whether binding specificity can be predicted from the PDZ domain's primary sequence, arises. The analysis by Tonikian *et al.* [7] established a predictive correlation between the domain sequence and binding specificity in organisms from worm to human. Most recently, Ernst and colleagues [8] found that ligand binding capability is inherent to the PDZ domain, and mutated variants can support specificities that do not exist in nature, suggesting that this structural and functional flexibility could be exploited to facilitate rapid rewiring of protein-protein interaction networks during evolution [9]. These significant findings represent a step toward the possibility of inferring protein interactions directly from a genome's sequence. The ability to accurately predict domain binding specificities from primary sequence in general, and for the PDZ domain in particular, would provide yet another step in that direction.

Addressing this problem, the DREAM (Dialogue for Reverse Engineering Assessments and Methods) Consortium issued a Peptide Recognition Domain Specificity Prediction Challenge. A part of this challenge was to predict position weight matrices (PWMs) that describe the specificity profiles of five PDZ domains to their target peptides. These test cases were modeled on the ERBB2IP-1 (Erbb2 interacting protein) wild type protein, each with multiple different mutations. The domains were examined experimentally using phage-displayed random peptide libraries, a powerful tool to elucidate domain specificity. The experiments determined short linear peptide fragments that bind each of the PDZ domains in question. The resulting binding patterns, represented as PWMs, were withheld as the “gold standard” to evaluate the challenge submissions.

Our approach, based on the experimental data set of Tonikian *et al.* [7] profiling single-mutant PDZ binding specificities, aimed at generalizing the effects of single point mutations on binding preference to multi-mutant sequences. To predict the challenge PWMs, we combined linear regression-based prediction for ligand positions whose specificity is known to be determined by relatively few PDZ domain positions, and single-mutant PWM averaging for all other ligand columns. Our resulting DREAM4 submission was the winning entry, obtaining better predictions than the next competing entry on four of the five test sequences. With the benefit of the gold standard PWMs now available, we determined that our method was close to the best possible combination of regression and averaging based predictions. Finally, we found that basing one's predictions on the domain-specific single-mutant data was more beneficial than following a general PDZ-ligand binding model such as that of Chen *et al.* [10].

## Results

### Predicting PWMs for ERBB2IP-1 mutants

**Figure 1:**
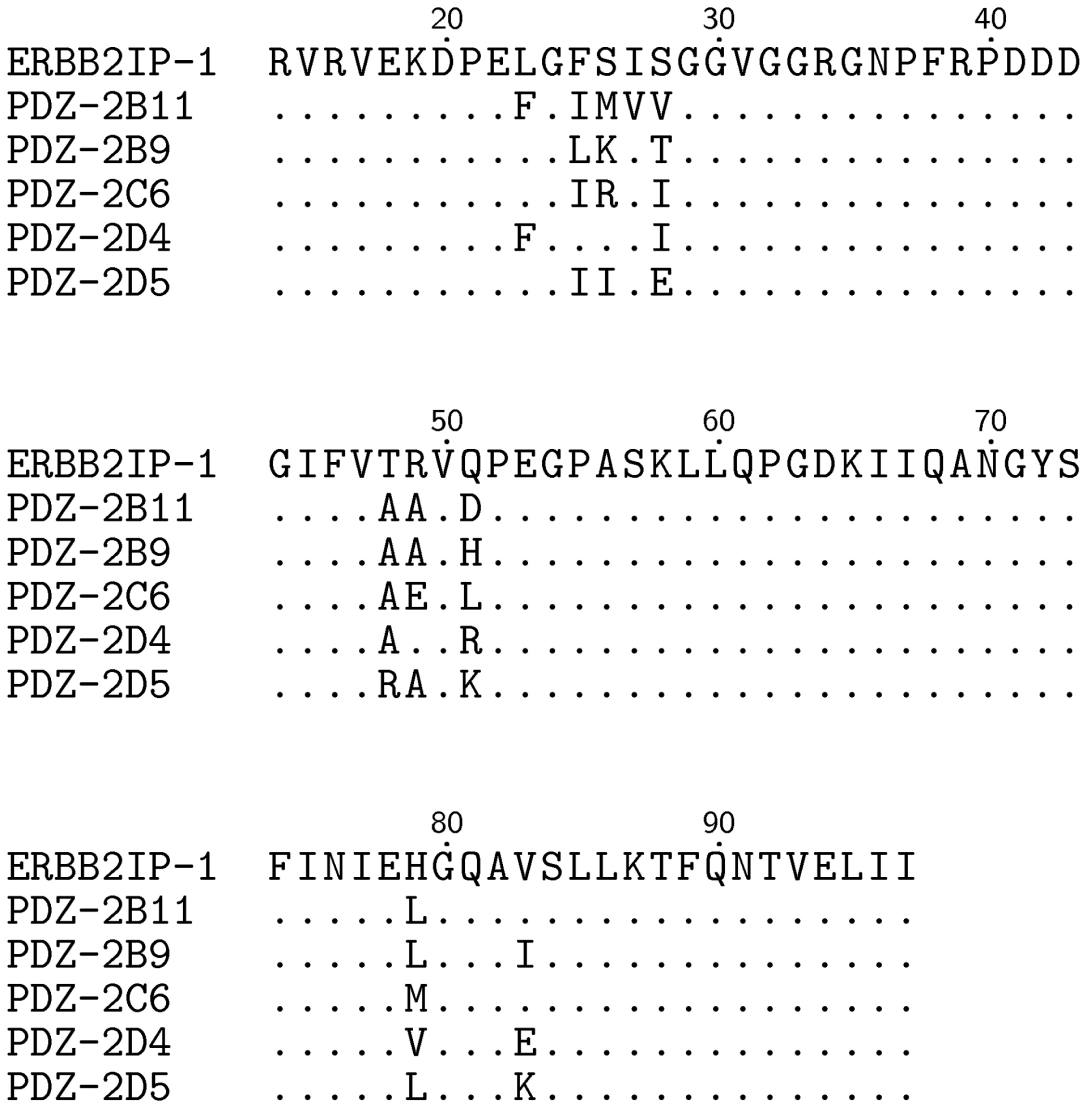
Wild type and challenge PDZ domain sequences. The top line lists the full amino acid sequence for the wild type ERBB2IP-1 PDZ domain. The following lines show mutations for the five test sequences. Amino acid numbering follows Tonikian *et al.* [7]. Graphics were generated using TEXshade [17].

The DREAM4 PDZ-peptide recognition challenge consisted of predicting binding specificities for five multi-mutant ERBB2IP-1 sequences (Figure 1). The PWMs predicted by our method for these sequences were composites resulting from two different approaches. The columns corresponding to ligand positions 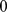 (ligand C-terminal position), 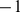, 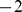, and 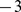 were predicted using a regression approach, and the remaining columns by clustering and averaging of corresponding PWM columns in the training set (see Methods). This choice was made following an observation by Tonikian *et al.* [7] that only a subset of the positions mutated in the ERBB2IP-1 protein affected binding preferences for ligand positions 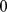, 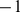, 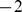, and 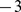. Thereby, we were able to reduce the regression's dimensionality and decrease the risk of overfitting. The remaining ligand positions have shown significantly less specific binding preferences, and were affected by a greater number of the mutated PDZ positions [7]. We chose to average the corresponding single-mutant PWM columns in deriving their multi-mutant profiles.

**Figure 2:**
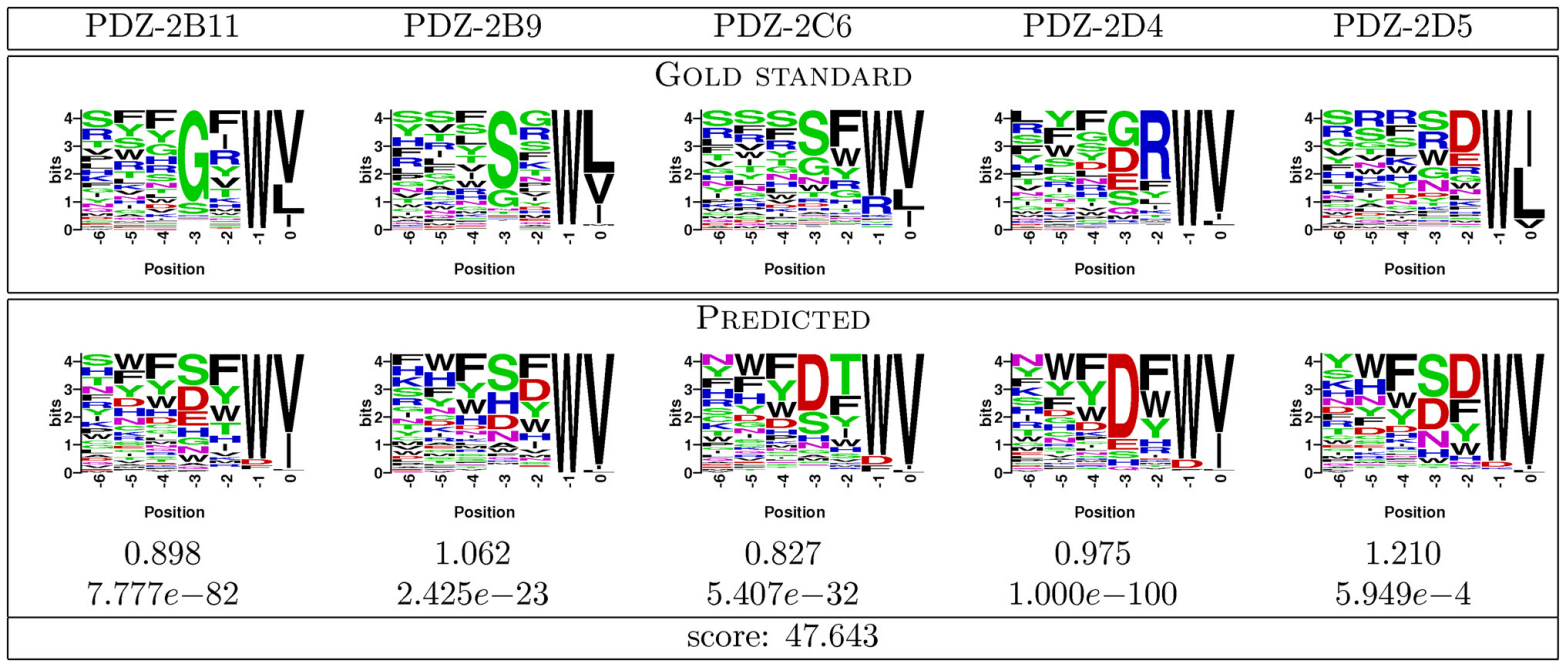
Gold standard and our predicted PWMs for the five multi-mutant proteins in the DREAM4 PDZ challenge. Top panel shows PWMs resulting from the phage display screening. Bottom panel shows our predicted PWMs together with Frobenius distances when comparing each prediction to the gold standard PWM, as well as the corresponding P-values. The final prediction score, a log-transformed “average” of the P-values for the five domains and the basis of the final challenge rankings, is shown on the last line.

Our resulting binding specificities for the multi-mutant test PDZ domains together with their gold-standard binding profiles withheld during the competition, are shown in Figure 2. Each submitted entry was compared with the corresponding gold standard PWM using the Frobenius norm. Then, individual P-values, defined as the probability that a random PWM has the same or smaller Frobenius distance to the measured PWM, were computed (and capped at 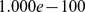). The final challenge score was indicative of the overall significance of the results, and a unit increase for one prediction over the other reflected an average one order of magnitude P-value improvement (see Methods). The individual domain P-values, predicted by our method, ranged between 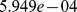 and 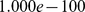. The final resulting score was 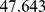, and our method was declared the PDZ challenge winner. By comparison, the next best entry had the final score of 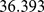, and worse individual Frobenius distances for four of the five test cases.

The high statistical significance of our predictions was largely driven by our method's ability to correctly recapitulate the highly specific ligand positions 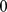 and 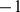 (see Figure 2). Indeed, if one were to predict PWMs such that the canonical tryptophan in position 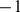 and valine in position 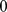 were each assigned unit probability, leaving all other columns uniform (“canonical” predictor), the final score would be 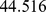. Our method predicted a very dominant tryptophan preference in position 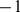 for all five PDZ mutants, and a strong preference for valine or leucine in ligand position 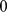; the exception here was test case PDZ-2D5, for which we incorrectly predicted valine instead of leucine / isoleucine, resulting in a far worse P-value than for the rest of the domains. Moreover, our ability to capture partial preferences of the less specific ligand positions, such as phenylalanine in position 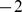 of PDZ-2B11, contributed to our high scores beyond what is achievable with only predicting canonical amino acids for ligand positions 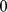 and 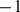. Indeed, our P-values were better than those of the “canonical” predictor by three orders of magnitude on average.

### Combining regression- and average-based PWM predictions

Our DREAM4 entry combined PWM columns predicted by a regression-based approach with columns obtained using a PWM averaging-based approach. With the benefit of published phage display derived PWMs for the five test mutant PDZ domains (Figure 2), we were able to assess our particular combination of the two methods for groups of columns in ligand binding sites. We examined eight alternatives in all, predicting varying numbers of columns closer to the ligand C-terminal position with the regression-based predictor, denoted 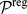, and predicting the remaining columns by the PWM single-mutant averaging-based predictor, denoted 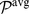. We also considered predicting entire PWM profiles using 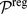 and 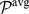 in turn for all columns. The resulting distances to the experimentally-derived PWMs, their corresponding P-values and scores are listed in Table 1. The best combined predictor, which used 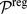 predictions for columns 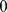, 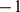, and 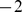, obtained the final score of 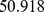, and found the lowest Frobenius distances for four of the five test sequences. Our DREAM4 submission, which differed in that column 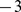 prediction was replaced with that of 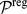, had the second overall result. As shown by Tonikian *et al.* [7], ligand position 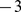 makes contact with seven of the mutated PDZ positions; it is likely that not enough data was available to train the regression-based method and avoid overfitting when predicting the specificities for this position. Interestingly, the other combinations we considered in Table 1, including the simplest predictor, which clustered and averaged single-mutant PWMs for all ligand positions, obtained better final scores than all other competing DREAM4 entries.

**Table 1:** Prediction results for combinations of averaging-based and regression-based PWM columns.

PWM	PDZ-2B11	PDZ-2B9	PDZ-2C6	PDZ-2D4	PDZ-2D5	score
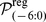	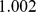 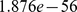	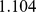 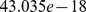	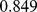 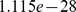	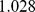 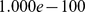	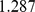 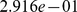	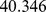
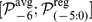	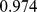 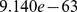	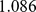 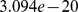	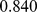 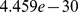	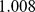 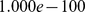	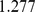 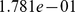	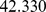
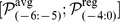	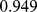 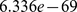	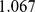 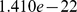	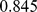 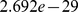	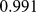 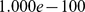	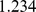 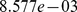	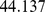
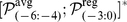	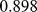 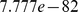	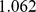 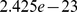	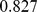 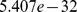	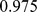 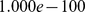	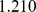 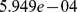	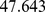
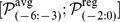	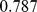 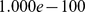	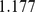 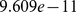	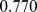 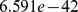	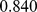 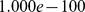	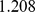 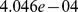	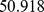
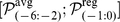	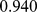 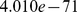	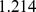 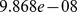	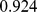 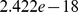	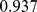 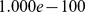	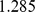 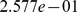	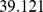
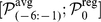	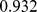 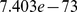	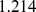 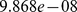	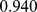 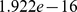	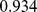 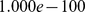	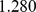 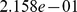	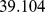
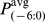	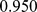 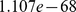	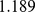 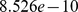	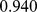 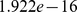	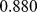 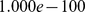	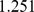 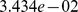	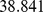

Each line corresponds to a PWM (with Frobenius distance to the experimentally-derived PWM, the P-value and score), derived as a combination of columns from the averaging-based predictor 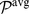 and the regression-based predictor 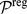; columns predicted by each method are indicated as subscript ranges. As elsewhere in the text, column 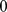 is the ligand C-terminal position. Lowest Frobenius distance for each challenge sequence is highlighted in bold, and our DREAM4 submission is denoted by 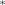.

### Comparison with a universal PDZ domain specificity model

The specifications of the DREAM4 challenge and public availability of ERBB2IP-1 single-mutant phage-display data have allowed us to design a method that uses such data in predicting binding specificities of multi-mutant domains. Alternatively, the binding specificity of a query sequence can be deduced from a universal PDZ domain family model. Arguably, such a model, trained using a much larger and more diverse data set, could potentially better depict subtle sequence-related specificity determining features. In the following, we assessed the performance of a general model of PDZ domain selectivity, recently introduced by Chen *et al.* [10], on single- and multi-mutant ERBB2IP-1 sequences, and compared it with ours.

**Figure 3:**
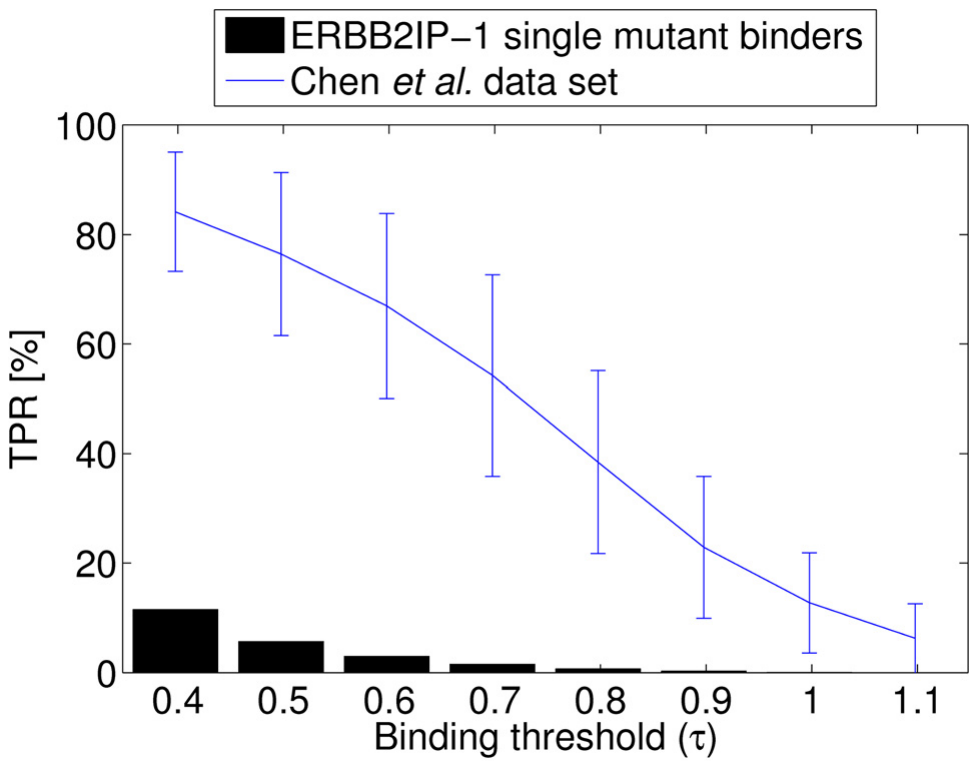
Chen *et al.* model performance on the ERBB2IP-1 single mutant data. True positive rate produced by the binary model of Chen *et al.* [10] for predicting ERBB2IP-1 single-mutant binders [7] is compared to the rate Chen *et al.* report for their data set [10]. The rate is profiled for a range of the binding threshold, 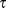.

We first examined the Chen *et al.* model predictions in identifying binders for single-mutant PDZ domains. It is reasonable to assume that a method should perform well on the single-mutant data before attempting to predict binding preferences for multi-mutant domains. We used the binary model of Chen *et al.* [10], trained on a quantitative PDZ domain interaction data set [6] and using 100 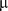
M dissociation constant as the threshold for defining an interaction. The binary model was chosen for evaluation since, as noted by the authors, it performed better when predicting novel interactions. Surprisingly, though, the results of predicting binders for single-mutant PDZ domains were very poor. As shown in Figure 3, the true positive rate profiled across the relevant model threshold (see [10] for details) was much lower than the comparable rate Chen *et al.* observed for their data set.

**Figure 4:**
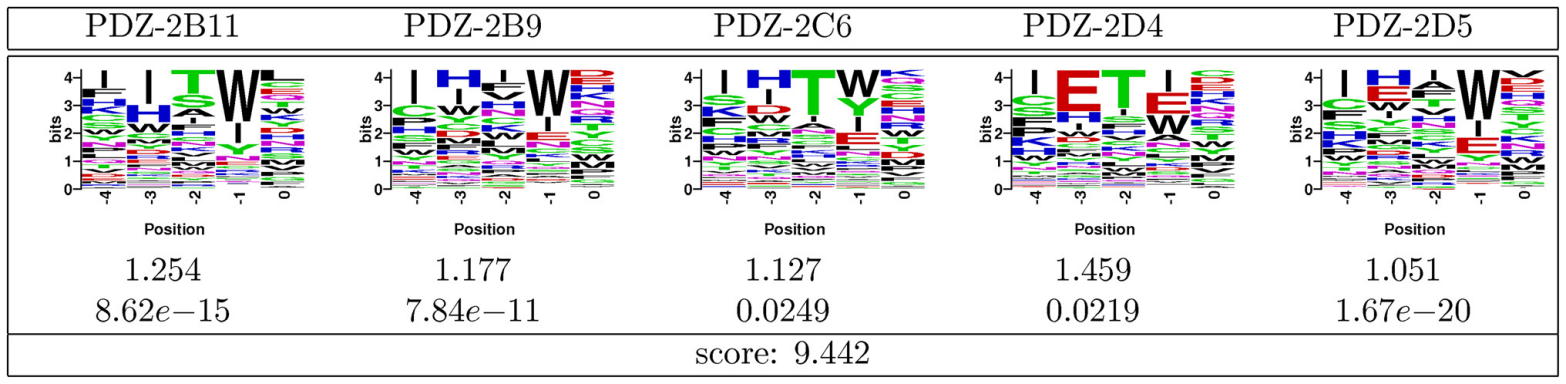
Performance of the Chen *et al.* model in predicting PWMs for the DREAM4 PDZ challenge sequences. PWMs generated using the binary model of Chen *et al.* [10] after converting model scores to Boltzmann probabilities (with the temperature parameter set to 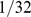). Individual Frobenius distances to the gold standard PWMs and their corresponding P-values as well as the overall resulting prediction score are listed.

We then derived PWMs for the five multi-mutant PDZ domains from the model of Chen *et al.* by converting their model scores into Boltzmann probabilities. In line with the low true positive rate for single-mutant ligand binding, the predicted multi-mutant PWMs were poor as well. Only three of the five test cases (Figure 4) showed significant similarity to the gold standard PWMs, obtaining a collective final score of 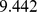, much lower than any of the scores in our combined model (Table 1). These results demonstrate the benefit of training a predictor for a specific PDZ domain (e.g, ERBB2IP-1 ), when feasible, on a well-selected data set, as opposed to using a single model for an entire domain family. It is conceivable, though, that the predictions of the Chen *et al.* model may be improved by the inclusion of additional information, and, in particular, the single-mutant phage-display data, in their training set.

## Discussion

We have presented a method for predicting PDZ domain binding specificity, used in the DREAM4 peptide recognition domain challenge to determine ligand binding profiles of five multi-mutant ERBB2IP-1 PDZ domains. Though the issued challenge focused on a very specific and well-defined problem, a paradigm similar to ours can be adapted for broader usage and, specifically, any domain, or domain family, for which multiple PWMs have been experimentally determined and, preferably, a set of interacting positions identified.

While our method was the winning entry of the DREAM4 challenge and performed very well on the defined task, improvements can be made. First, following the challenge specifications of predicting PWM models, we assumed positional independence between columns in the ligand, a potentially simplifying assumption. With the recent publication of binding data for a large set of ERBB2IP-1 multi-mutant domains [8], a more thorough examination of this aspect is possible. In particular, such data sets would permit training of more sophisticated machine learning-based specificity predictors that allow for modeling pair-wise or even higher order positional dependencies, both in the ligand and in the domain.

Second, the predictions for a few positions, mainly the low specificity N-terminal ligand positions, can be significantly improved. Notably, our training set appeared so limited that for a few such positions lower Frobenius distances to the gold standard PWMs would have been obtained by predicting a uniform PWM column rather than the ones derived by either the regression or average-based approaches. With the availability of a larger and richer training set, this shortcoming can probably be ameliorated. Nonetheless, studying the binding profiles at these low specificity positions raises the question of whether these differences are meaningful at all. While the Frobenius norm is a well-established mathematical metric, it does not differentially score close-to-background and highly specific positions, an approach that might be beneficial for cases like the PDZ domain, where the degree of specificity in the ligand columns varies greatly. A biologically-motivated function, such as the Bayesian Likelihood 2-Component (BLiC) [11] function, might better highlight the essential differences, and similarities, between a given pair of PWMs.

## Methods

### Training data

Tonikian *et al.* [7] have studied the effect of point mutations on binding preferences of the ERBB2IP-1 PDZ domain. They considered mutations at ten binding site domain positions (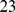, 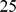, 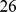, 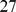, 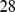, 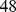, 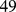, 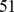, 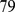, 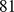), and for each such position (e.g. 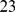), they created a single-mutant variant, substituting the wild type amino acid (L) with other amino acids (F, I, V) commonly found at that PDZ position. In all, 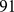 ERBB2IP-1 binding site point mutations were characterized, individually, using C-terminal phage-displayed libraries [7], and sequences of thousands of peptides that bind to these single-mutant PDZ domains, as well as wild type ERBB2IP-1 , had been determined.

Following Tonikian *et al.*, we generated a position weight matrix (PWM) for every single-mutant PDZ variant based on its set of binding peptides, adding no pseudo-counts and correcting for codon bias by dividing observed amino acid frequencies by their expected frequencies in the NNK codon set [7]. We then utilized the resulting PWMs to train our models. Note that since the PDZ challenge focused solely on ERBB2IP-1 mutants, we only considered ERBB2IP-1 -related sequences in our training set and deliberately disregarded similar data for other PDZ domains available in databases such as PDZBase [12] or DOMINO [13].

### Predicting PWMs

The DREAM4 PDZ-peptide recognition challenge was comprised of five multi-mutant ERBB2IP-1 sequences (Figure 1), each containing between six and nine mutations with respect to the wild type domain, from within the set of 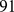 single point mutations characterized by Tonikian *et al.* [7]. Our method generalized the effects of single point mutations in PDZ domains on binding preference, as measured experimentally, to multi-mutant sequences. To that end, we experimented with two different approaches: regression- and PWM averaging-based prediction.

#### Regression-based PWM columns

In defining the prediction model, we needed to identify the PDZ positions that affect binding specificity of every ligand position. Importantly, limiting sets of interactions between a ligand position and PDZ domain residues necessarily reduces the complexity of any potential predictor. We were able to restrict the set of interacting PDZ positions for ligand positions 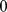 (ligand C-terminal), 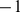, 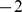, and 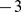 following Tonikian *et al.*, who have shown that binding preferences at these positions are determined, in large part, by mutations at PDZ positions 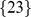, 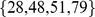, 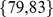, and 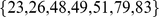, respectively. For the remaining ligand positions, with no such experimentally restricted set of interactions, we considered the amino acids in all ten binding site positions.

**Figure 5:**
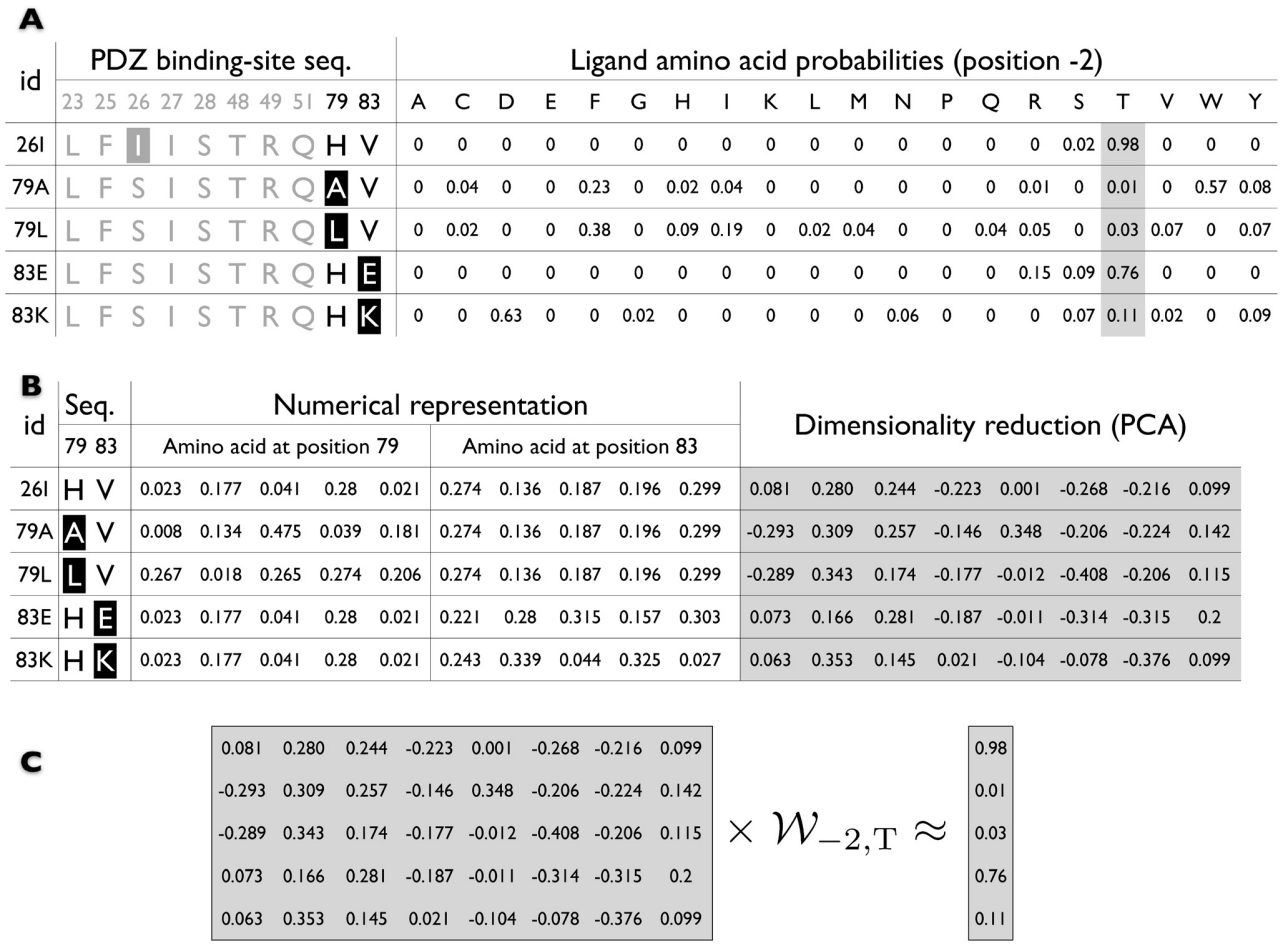
Regression-based specificity prediction. (**A**). Shown on the left are PDZ binding site single-mutant sequences. Positions, not relevant for predicting a particular ligand position (illustrated here for position 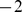 and shown in grey), are disregarded. The mutated amino acids are highlighted. Shown on the right are single-mutant specificities corresponding to ligand position 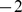. (**B**). Subsequences at relevant specificity determining positions are converted into numerical vectors, and dimensionally reduced using PCA. (**C**). The resulting vectors, along with the corresponding per-amino acid probabilities (here, for amino acid threonine (T)), define a regression problem. The coefficients obtained by solving such regression problems are used to predict the probability of each amino acid at a given ligand position.

For a particular ligand position, we represented each amino acid among the set of interacting PDZ positions (Figure 5A) as a five-dimensional vector, derived by projecting a corresponding high dimensional physical-chemical property vector onto the five most significant principle components [14], and taking the modulus of the resulting values. Each subsequence was then encoded as a concatenation of such five-dimensional vectors (Figure 5B, left). Such a representation has been shown useful in various binding prediction scenarios (e.g., [15]). Next, principal component analysis (PCA) was applied to further reduce the input space dimensionality, discarding components along which the variance of the data was less than 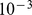 (Figure 5B, right). The per-position data matrix, consisting of these PCA-based single-mutant and wild type vectors, and the PWM-derived probabilities for each amino acid at the corresponding position defined a set of regression problems (Figure 5C). Given a query PDZ sequence, the regression coefficients obtained by solving these problems could be used to predict a “pseudo” probability for each amino acid at each ligand position. For the final regression-based PWM, denoted 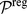, we replaced negative entries with zeros, and normalized each per-position vector to sum to one.

#### Averaging-based PWM columns

**Figure 6:**
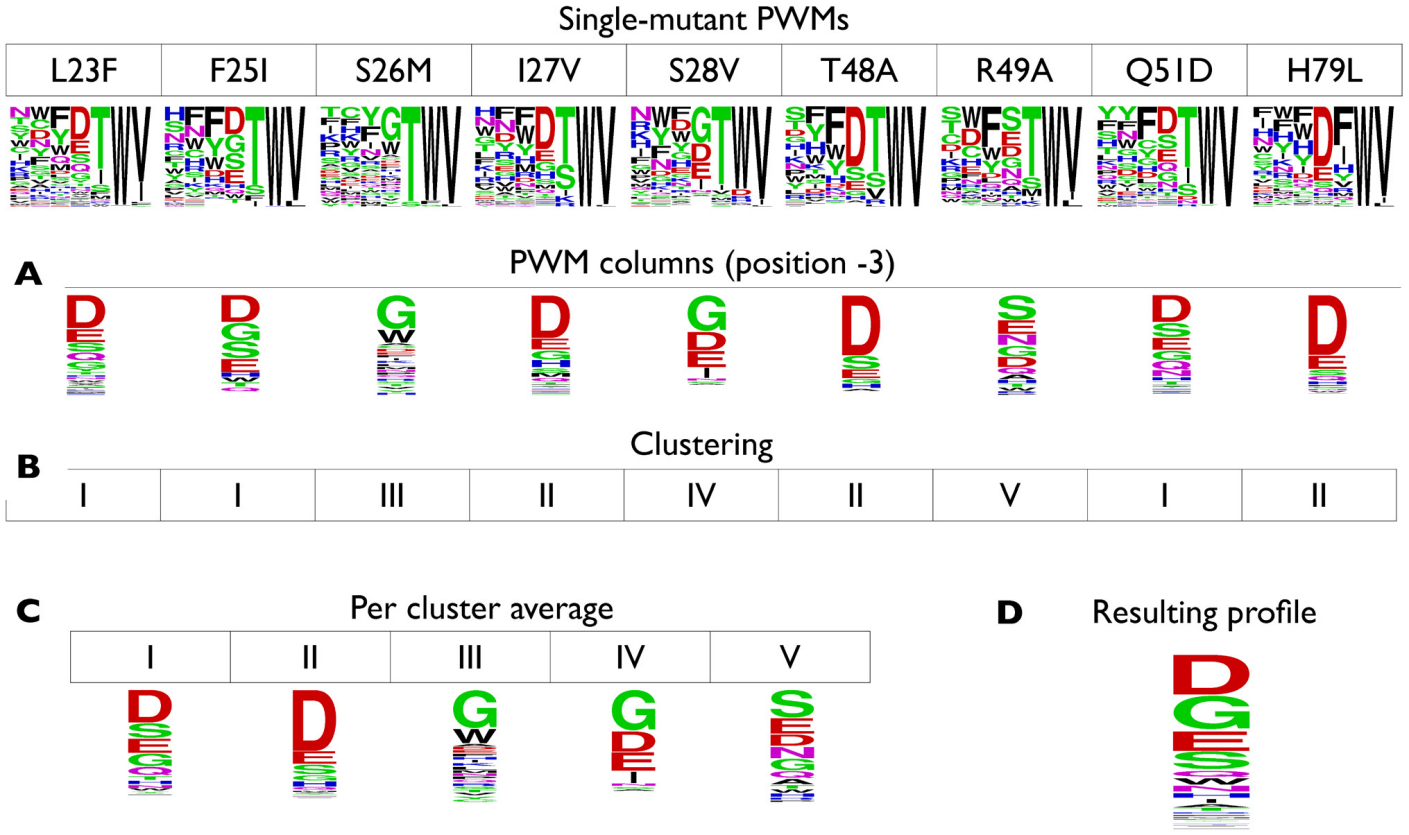
PWM averaging-based specificity prediction. For each multi-mutant sequence and ligand position (shown here for PDZ-2B11 and position 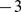), the corresponding single-mutant PWM columns were extracted (top panel and **A**) and clustered (**B**). The predicted profile (**D**) is then defined as the average of the per cluster representative vectors (**C**).

We speculated that, in some cases, the regression approach might lead to over-fitting and, therefore, considered a supplementary, more “conservative” approach denoted 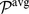, directly based on averaging PWM columns. While the regression-based predictors attempted to infer physical-chemical “rules” of binding preferences and, to this end, incorporated information from all available single-mutants, the average-based approach considered a smaller, but perhaps more relevant, set of single-mutants. In particular, for a given DREAM4 challenge sequence and for each ligand position, we extracted the per-position amino acid probability vectors from the corresponding point mutant PWMs (Figure 6 top and A). Since point mutations mostly have a local effect on ligand binding preferences, many of these vectors likely reflect the binding preferences of the wild type domain. We therefore grouped the vectors into clusters and averaged the cluster representative vectors in an attempt to avoid biasing our amino acid preferences toward the wild type. Specifically, we partitioned the input vectors into five clusters, using complete linkage clustering with Euclidean distance as a similarity measure, and computed the average over all vectors in a cluster as its representative (Figures 6B,C). Finally, we averaged these cluster representatives to obtain the average-based PWM, 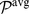 (Figure 6D).

### DREAM4 scoring metrics

The similarity between a computationally predicted PWM and its gold standard, experimentally-determined counterpart was judged using the Frobenius norm, computed as follows:
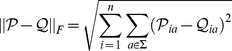
where 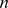 denotes the number of columns (that is, ligand positions) in PWMs 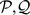, and 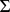 the alphabet, here the set of 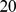 amino acids. To estimate a target-specific P-value for a given Frobenius distance 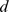, the DREAM4 organizers simulated an empirical frequency distribution of Frobenius distances between the experimental PWM and 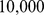 randomly generated PWMs, and fit it to stretched exponential functions, with different parameters to the right and left of the mode of the distribution, as previously described [16]. These functions were then used to compute the probability of obtaining, by chance, a distance equal to or better than 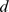. Finally, the overall DREAM4 score was defined as the average, over the five PDZ target sequences, of the negative 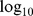-transformed P-values, where larger scores indicated greater statistical significance of the prediction.

### Chen *et al.* model: a sequence-based PDZ specificity predictor

Chen *et al.* [10] built a model to predict binding for arbitrary PDZ domain-peptide complexes, using their primary sequences only. The model identified, based on structural information, 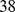 potentially interacting position pairs, involving 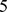 C-terminal ligand positions and 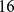 PDZ domain positions (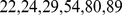 as well as the ten binding site positions, listed above, used by Tonikian *et al.* [7]). Specifically, ligand positions 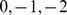 and 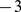 were coupled with numerous (between seven and ten) PDZ positions each, creating a very dense interaction network. Note that, in contrast, the ERBB2IP-1 domain-ligand interaction network observed experimentally by Tonikian *et al.* is much sparser, with as few as one or two interactions for some ligand positions.

For each of the 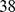 potential interaction pairs, the model of Chen *et al.* [10] inferred a score matrix, indexed by, and assigning a score to, each combination of amino acids at the corresponding PDZ-ligand positions. These pair-wise scores were then summed up to give a final binding score; when this score was greater than some defined threshold, the PDZ domain was predicted to bind the peptide.

To predict binding specificities for a given ERBB2IP-1 multi-mutant sequence and some ligand position, we had to convert model scores to PWM probabilities. First, we summed the 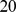-entry columns indexed by the amino acids in the multi-mutant PDZ sequence and contributing to interactions with the ligand position under consideration. We then converted these column scores to Boltzmann probabilities using various system “temperatures”, and report the results for a temperature setting of 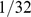, which obtained the best overall DREAM4 score. Note that the Chen *et al.* model is applicable only to the five C-terminal ligand positions and, therefore, uniform amino acid preferences were assumed for the remaining positions.
